# On‐Demand Coalescence and Splitting of Liquid Marbles and Their Bioapplications

**DOI:** 10.1002/advs.201802033

**Published:** 2019-03-14

**Authors:** Ben Wang, Kai Fung Chan, Fengtong Ji, Qianqian Wang, Philip Wai Yan Chiu, Zhiguang Guo, Li Zhang

**Affiliations:** ^1^ Department of Mechanical and Automation Engineering The Chinese University of Hong Kong Hong Kong China; ^2^ Department of Biomedical Engineering The Chinese University of Hong Kong Hong Kong China; ^3^ Chow Yuk Ho Technology Centre for Innovative Medicine The Chinese University of Hong Kong Hong Kong China; ^4^ Department of Surgery The Chinese University of Hong Kong Hong Kong China; ^5^ State Key Laboratory of Solid Lubrication Lanzhou Institute of Chemical Physics Chinese Academy of Science Lanzhou 730000 China; ^6^ Hubei Collaborative Innovation Centre for Advanced Organic Chemical Materials and Ministry of Education Key Laboratory for the Green Preparation and Application of Functional Materials Hubei University Wuhan 430062 China; ^7^ T Stone Robotics Institute The Chinese University of Hong Kong Hong Kong China

**Keywords:** laboratory automation, liquid marbles, manipulation, miniature bioreactors, nanoparticles

## Abstract

Coalescence and splitting of liquid marbles (LMs) are critical for the mixture of precise amount precursors and removal of the wastes in the microliter range. Here, the coalescence and splitting of LMs are realized by a simple gravity‐driven impact method and the two processes are systematically investigated to obtain the optimal parameters. The formation, coalescence, and splitting of LMs can be realized on‐demand with a designed channel box. By selecting the functional channels on the device, gravity‐based fusion and splitting of LMs are performed to mix medium/drugs and remove spent culture medium in a precise manner, thus ensuring that the microenvironment of the cells is maintained under optimal conditions. The LM‐based 3D stem cell spheroids are demonstrated to possess an approximately threefold of cell viability compared with the conventional spheroid obtained from nonadhesive plates. Delivery of the cell spheroid to a hydrophilic surface results in the in situ respreading of cells and gradual formation of typical 2D cell morphology, which offers the possibility for such spheroid‐based stem cell delivery in regenerative medicine.

## 1 Introduction

Liquid marbles (LMs) are mainly composed of liquid droplets that are encapsulated and stabilized by solid micro or nanoscale lyophobic particles at the spherical liquid–air interface.^1^ They show extreme mobility, elasticity, and stability on both hydrophilic and hydrophobic surfaces. LM has attracted increasing attention in the past decade due to its potential applications in gas sensing,^2^ adhesive materials,^3^ microchemical reactors,^4^, ^5^, ^6^, ^7^, ^8^, ^9^ water pollution indicator,^10^ and so forth.^11^, ^12^, ^13^, ^14^, ^15^ Many types of liquid, with reactants and precursors dissolved inside, can be encapsulated by the particle shell to obtain LMs with a wide range of volumes in microliter scale.^16^, ^17^, ^18^, ^19^, ^20^ In contrast to the naked liquid droplets, the as‐obtained LMs can be deemed and manipulated as nonsticky materials, which dramatically decrease the surface free energy and boost the handleability of the tiny drops. Moreover, the LMs were demonstrated to possess a significantly longer lifetime than that of the naked droplets.^21^ The lifetime is related to many aspects, like particles size, surface chemistry, and the external conditions.^21^, ^22^, ^23^ The manipulation of single LMs, by the mechanical force,^24^, ^25^ magnetic locomotion^26^, ^27^, ^28^ and light‐driven motivation^29^, ^30^ have been developed in the past decade. Benefiting from the single LM manipulation and liquid control, the coalescence of the well‐prepared LMs can mix the precursors in a precise amount and the outer particle shell, a protective screen, can avoid the inner liquid loss and protect the inner liquid from being contaminated by the external environment. The splitting process allows people to isolate the useless wastes after each stage of reactions, especially for the splitting of metabolites during the long‐time culture of cells. The coalescence and splitting of two or multiple LMs are the key factors to realize the extensive application in the precise and trace amount reactions in both stoichiometric chemistry and biomedical applications such as cell culture.

To date, however, the interaction and coalescence/splitting of LMs are not yet be fully studied and remain in an early stage with few deeper diggings in both the methodology, theory, and applications. The coalescence of LMs was conducted mainly through a magnetic attraction for opening and closing of the particles shell of LM, or manual injection with a microsyringe to achieve the LM‐based reactions.^31^, ^32^ In addition, acoustic levitation strategy has been applied to realize the manipulation, and the opening and closing of the levitated LMs could be achieved from variation of the sound intensity and frequency.^33^, ^34^ However, such a method is irreversible, power consuming, and generally limited to light and small LMs. The sustained shaking of the LMs is inevitable, which may interfere the inner liquid reactions especially for the cell activities in the biomedical applications. Recently, researchers have applied the LM to culture cells in the 3D format by manually rolling the medium on a hydrophobic powder.^35^, ^36^, ^37^, ^38^, ^39^ However, the proposed method applied a tedious manual culture process and the sustained supply of nutrients and medium during medium renewal was difficult to achieve,^40^ which hampered the long‐time culture of the 3D cell spheroid.

Here, we introduced a centrifugal force‐ and gravity‐based method to achieve rapid formation, coalescence, and splitting of the LMs for 3D culture of stem cells in vitro, drug/nutrient addition, and waste medium removal using a highly automated process to ensure that the cellular microenvironment of such 3D cell culture was maintained under optimal conditions for long‐term. An additional benefit of the method is the versatility for various kinds of LMs. The method was systematically investigated to obtain optimal parameters to realize both coalescence and splitting. The size of the cell spheroid generated from the system could be controlled within a wide range, from the micrometer to sub‐millimeter scales. The prepared cell spheroids showed highly compact cell‐to‐cell cross‐contacts and formed the in vitro tissue, which is more valuable for the simulation of a real in vivo tissue than the 2D cell monolayers. More importantly, the obtained stem cell spheroid (SCS) from the coalescence and splitting system (CSS) showed much higher cell viability than that of the traditional culture method, which is probably attributed to the high gas permeability of the LMs. The CSS shows potentials as a bioreactor for controlled long‐time in vitro tissue cultures used in regenerative medicine, drug screening, and tissue engineering.

## 2 Results and Discussion

**Figure 1:**
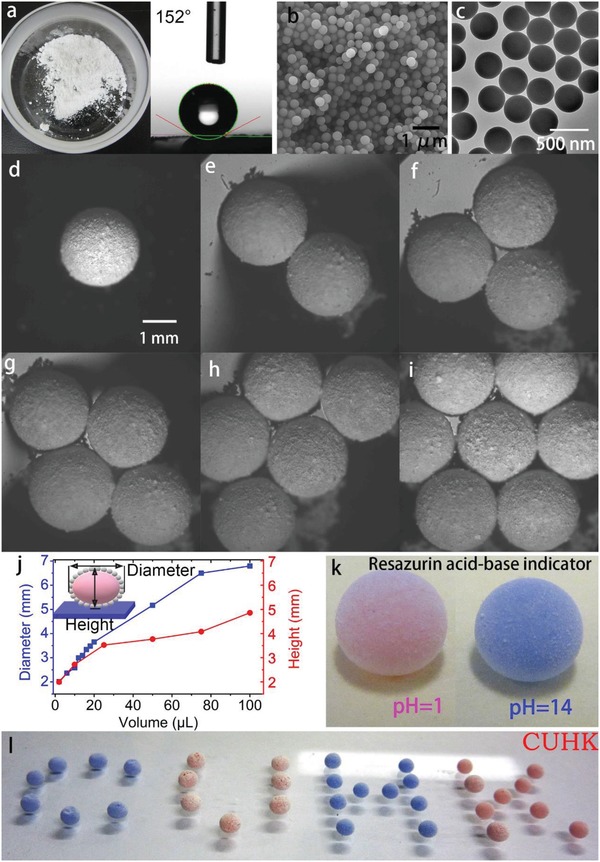
a) Optical images showing the silica particles and the contact angle of the sample. b) SEM image showing the surface morphology and particle size of the SiO_2_ particles. c) TEM image showing the surface morphology and particle size of the SiO_2_ particles. d–i) Self‐assembly of LMs (6 µL) on the water surface: d) one LM, e) two LMs, f) three LMs, g) four LMs, h) five LMs, and i) seven LMs. j) Relationship between the diameter, height, and the inner liquid volume of a LM. k) Optical image showing the colors of the LMs with resazurin aqueous solution at different pH values. l) Optical image showing several LMs with resazurin aqueous solution arrange to the word “CUHK”. The letter “C” and “H” were composed of pH = 14 ammonium hydroxide solution and the letter “U” and “K” were composed of pH = 1 hydrochloric acid solution.

The SiO_2_ microspheres were prepared by hydrolysis and condensation of TEOS, followed with grafting of a layer of trimethoxymethylsilane. The resultant powders are white and water‐repellent with a water contact angle (5 µL) of ≈152° (**Figure** **1**a). When a water droplet was dropped onto the bed of the powder, the droplet would be wrapped with the particles while rolling around due to the tendency to minimize the surface free energy, forming the so‐called liquid marble. The resultant LM is white, semitransparent, and sphere‐like with the SiO_2_ particles evenly distributed on the shell. The shell of millimeter‐sized LM is composed of spherical SiO_2_ particles with a uniform size distribution of about 300 nm as illustrated by scanning electron microscopic (SEM) image and transmission electron microscopic (TEM) image in Figure 1b,c. The chemistry of the particles was investigated by XRD diffractogram which suggests a broad peak located at 2θ = 22.9° (Figure S1, Supporting Information). The characteristic amorphous peak is attributed to silica according to the JCPDS data with card No. 01‐086‐1561. The volume of the LM can be precisely controlled in a wide range, from 0.5 to 100 µL. Figure S2 in the Supporting Information selectively gives the microscopic images of the LM with different liquid volumes. The diameter as well as the height of LMs increase with the increase of the inner liquid volume (Figure 1j). When the LMs were put into a large volume culture dish with water, the LMs tended to self‐assemble to form specific patterns on water which may attribute to the capillary force,^41^ as is shown in Figure 1d–i. The process was spontaneous and the geometry of the LM patterns varied with the increase of the LM numbers. Owing to the semitransparency of the LMs, the LMs can be applied as the sensor for gas detection. As shown in Figure 1k, LM with pH = 1 aqueous resazurin solution shows pink color whereas LM with pH = 14 aqueous resazurin solution shows a blue color. Figure 1l gives the demo of the LMs with different liquid pH values forms “CUHK” logo. The LMs show high stability on various kinds of surfaces/interfaces, such as hydrophilic surfaces, hydrophobic surfaces, and even aqueous solution surfaces. The LMs could reside on the surfaces/interfaces for a long period until the inner liquid is totally evaporated to form a “deflated ball”.

**Figure 2:**
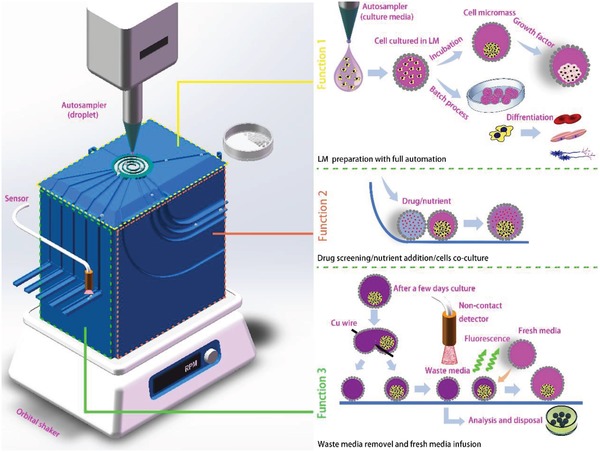
Schematic of the CSS, an on‐demand LM manipulation system, toward its application in the 3D SCS culture.

Taking the advantages of the gravity and centrifugal forces, we develop the automatic CSS (**Figure** **2**), via 3D printing of polylactic acid, to realize fabrication, coalescence, and splitting of LMs all‐in‐one by selecting the specific channels. The system includes, from bottom to up, the orbital shaker, core 3D printing device, detection sensor, spiral channel, and autosampler of the droplet. The top panel contains the central spiral groove required for the formation of LMs and the all‐round channels used for transporting and steering the LMs on the track. The side panels are functional channels required for the fusion process and the splitting process, respectively. Alternatively, they can be designed on‐demand. As illustrated in Figure 2, the CSS system could be used for in the cell growth related potential applications and the Function 1, 2, and 3 can be completed simply by selection of the channel from rotating the central spiral channel to match the transportation channels. The Function 1 is to realize the LMs autoformation via dropping of media with stem cells to hydrophobic powder at the central spiral channel and the centrifugal motion driven by the orbital shaker can make both the uniform coverage of powers on media and transportation of the as‐formed LMs to the destination. Compared with the conventional method to fabricate LMs by manual rotating on a dish, this method shows the high automation and uniformity of all the prepared LMs. The Function 2 is to coalesce the fresh‐formed LMs to make the bioreactions, such as nutrient addition, drug screening, coculture of different kinds of cells. Similar to the previous work, the gravity can be utilized to coalesce the LMs with the guidance of the channel.^42^ The direct addition of nutrient/drugs with a needle injection can also realize the mixing of bioreactant. However, the manual method is difficult for the operation of the microliter level droplet. More important, the ultimate capacity of the LM is limited by the outer hydrophobic particles. The shell can only tolerate slight expansion originated from the external addition, otherwise, the surface defects will generate, resulting in break up. The amount of media by the needle injection is therefore extremely confined. As for the coalescence‐based mixture, a large amount of media/bioreactant can be mixed into the original LM since the hydrophobic powder on the two LMs are sufficient to enwrap the coalesced marble without any surface defects. The Function 3 is to separate the formed LMs to realize the waste media removal and further analysis of the waste media after a certain period of culture. As the 3D cell spheroid has been formed inside the LM, the gravity‐driven cutting of the LM will separate the LM into two counterparts and the 3D cell spheroid will stay inside one of the sub‐LMs. The fluorescence sensor can be installed for screening in the outcome channel to inspect which LM has the spheroid. The LM without SCS can be screened for further analysis and other disposals. The LM with spheroid in it can be used to do another coalescence process with a LM of fresh media via the system. Each functional panel has several channels which are used for the LMs with different sizes/volumes.

## 3 Formation

**Figure 3:**
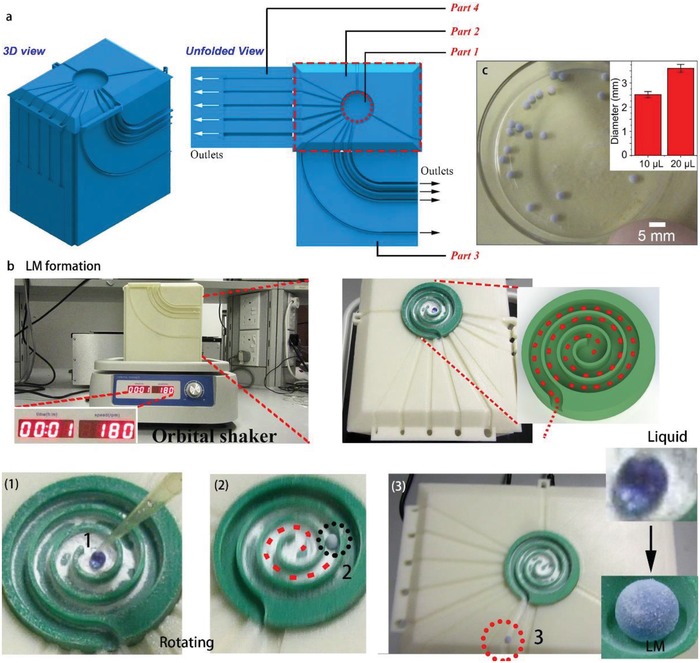
a) Schematic of the CSS with 3D view and unfolded view. b) Photograph showing the CSS with an orbital shaker underneath and the successive images of the LM formation process after a liquid droplet is added on the center of the spiral channel. c) Optical image of the as‐prepared LMs (10 µL). The inset shows the statistical average diameter of the as‐prepared LMs with high consistency.

The 3D view and unfolded view of the device is shown in **Figure** **3**a. The central spiral groove is moveable so as to select the specific pathway for realizing on‐demand functions. LM formation was completed by simply dropping the liquid in the center of the spiral channel followed by turning on the orbital shaker, as shown in Figure 3b. After the shaker was turned on with a final stable rotating speed of 180 rpm, the liquid droplet on the bed of the SiO_2_ quickly formed an LM. The centrifugal force‐driven circular motion with a gradually increasing radius ensured both the uniform coverage of powders on the medium and transportation of the as‐formed LMs to the ultimate destination. Figure 3c shows the photograph of a batch of as‐prepared LMs with uniform size and acceptable error range as indicated by the inset at the top right corner. Compared with the conventional method to fabricate LMs by manual rotating on a dish, this method shows the potential for mass production of LMs with high uniformity in an automatic manner.

## 4 Coalescence

To coalesce the LMs, the liquid inside the LMs should overcome the energy barrier generated from the outer hydrophobic powder to get into contact with each other. The energy barrier between the LMs can hardly be overcome via normal compression because the shell of an LM is composed with multiple layers of silica nanoparticles. The hydrophobic layers of silica nanoparticles of two LMs prevent all of the formation of liquid bridges. (see Movie S1 in the Supporting Information). After liquid‐to‐liquid contact occurred, the coalescence of the LMs would be a spontaneous process since they always tried to reduce their total surface area so that the total surface free energy of the system would be reduced accordingly to realize a lowest energy state, i.e., quasi‐spherical shape (Figure S3, Supporting Information).

To investigate the coalescence caused by LMs impact, we first investigated the process of an LM impacting a solid substrate, which is also demonstrated by Marston and co‐workers.^43^ The substrate we used is a hydrophilic glass slide. As shown in Figure S4a in the Supporting Information, the still frames of speedy movies depict the integrated process of LM (6 µL) impacting a glass slide from different heights (from 2 to 7 cm). The LMs show repetitive bouncing on the glass slide with gradually decreased jumping heights. With the increase of the falling height, the jumping heights after the first surface collision also increased. When the falling height is within the range of 5–6 cm, the LM would split into two sub‐LMs with the upper sub‐LM always larger than the lower one. While the falling height is no less than 7 cm, the LM would collapse as soon as the LM collide the surface (within several milliseconds). In addition to the integral view of the whole dynamic impact process, the first collision between the LM and the surface was taken into consideration by enlarged successive images as showed in Figure S4b,c in the Supporting Information, with both side and bottom views (falling height is 3 cm, LM is intact during the entire process). As shown in Figure S4b in the Supporting Information, the liquid droplet was evenly wrapped in white SiO_2_ shell with multiple layers. After collision with the substrate, the LM showed a serious deformation and became pancake shaped within only 3 ms. The serious deformation and complicated flow inside the LM made a small number of the outer layer of hydrophobic particles without direct contact and trapping on the water–air interface be splashed out. The peeling off of the shell particles mainly occurred at the moments of either impact of LM with the substrate at the lowest pancake thickness at the border region of the LM (3 ms, Figure S4b, Supporting Information) or bounce process at the center of the LM (6.5 ms, Figure S4b, Supporting Information). The major part of the hydrophobic particles on the shell would make themselves adapt the shape variation of the inner droplet and surface area increase of the droplet. As is known, a droplet shows its lowest surface area at the spherical shape. The deformation of the LM made the surface area of the LM increase, therefore producing the defects, especially when the LM deformed into a pancake shape, as showed by side view and bottom view in Figure S4b,c in the Supporting Information. The LM showed pancake shape at the maximum deformation of the impact process and the diameter of the pancake shape is measured to be 3.8 mm, which is 1.4 mm larger than that of the 6 µL LM at its spherical stage (60% expansion on diameter, the second image in Figure S4c in the Supporting Information). Figure S4c in the Supporting Information gives the bottom view of the impact process at its spreading stage, the pancake shape at the maximum deformation, and the retraction stage. The increase of the surface area at the pancake state causes the shortage of the hydrophobic particles to have a full coverage of the droplet, making rich surface defects and bare regions.^44^ These surface defects may facilitate the coalescence process of the LMs during impact processes.

The surface defects and bare regions are highly dependent on the inner flow field of the LMs. The simulation results of the impact between a droplet and hydrophilic glass were presented (Figure S4d,e, Supporting Information). This simulation is established using the phase field method in COMSOL Multiphysics to investigate the velocity field during the impact process, where a droplet and surrounding air are modeled as two separate phases. We use 2D axisymmetric computational domain due to the axisymmetry of the whole process. The droplet with a radius of 1 mm is initially positioned at a 5 cm distance above the substrate with zero initial velocity. The droplet freely falls downward under the influence of the gravity force and reaches the substrate at an impact velocity. Open boundary conditions are applied at both the top and side, simulating an infinite domain. A wetted wall boundary condition is used for the substrate with a contact angle of 152°. The successive images in Figure S4d,e in the Supporting Information show the inner flow field variation tendency during the process of drop‐solid contact to pancake shape. The inner velocity field near the spherical edge shows increased intensity and finally reaches its maximum value at the pancake shape as the time elapse (Figure S4e, Supporting Information). Apart from the sharply increased surface area, the nonuniform flow field inside the droplet may also contribute to the generation of the surface defects.

**Figure 4:**
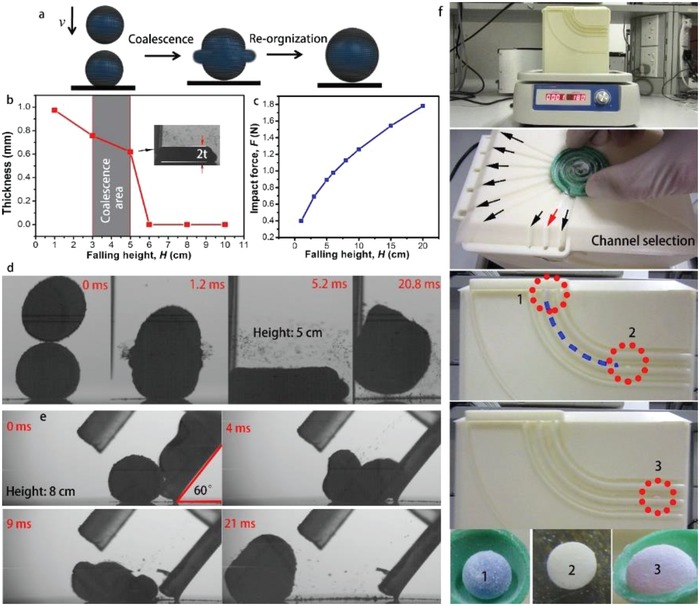
a) Schematic of the coalescence and reorganization process via collision. b) The relationship between the thickness of the pancake shape at the maximum deformation and the falling height of the upper LM. The inset shows the pancake shape at the maximum deformation. c) The relationship between the impact force between LMs and the falling height of the upper LM. d) Successive images showing the side view of the coalescence of two LMs with a falling height of 5 cm (also see Movie S3 in the Supporting Information). e) Successive images showing the side view of the coalescence of two LMs at a falling height of 8 cm with a tilt angle of 60° (also see Movie S4 in the Supporting Information). f) Photographs give the demo of the coalescence process of the LMs with the CSS.

To overcome the energy barrier caused by the outer layer of hydrophobic particles, we use the gravity‐induced collision to overcome the energy barrier and coalesce the LMs. As shown in **Figure** **4**a, one of the LM is resided on a glass slide and the other LM is released at a certain height right above the bottom one. When the height is too small (height = 1 cm, Figure S5, Supporting Information), the collision between the LMs could not produce sufficient surface defects to make the liquid‐to‐liquid contact occur. Then the LMs would be bounced away and the coalescence effect did not occur. When the height increased to a certain height, the coalescence behavior would occur. Figure 4d gives the LM impact at a height of 5 cm, both the top and bottom LMs were squashed by the collision and became pancake shaped at a maximum deformation (5.2 ms) as boxed by the blue line. The two LMs coalesced during the process and bounced off the substrate within 20.8 ms. As is shown in Figure S4 in the Supporting Information, the impact of LM could make the surface particles splash and the rearrangement of the surface particles makes the distribution of the particles uneven. The circinate sparse areas of the particles should appear on the surface of LMs and these areas were breakthrough for the instantaneous coalescence. As the height of the upper LM increased, the coalescence effect could not sustain due to the overlarge impact force, forming high‐adhesion wetting (Figure S5, Supporting Information). During the process of LM collision, the maximum deformation is an important aspect to measure the sustaining tolerance ability of the LM under collision. Here we use the high‐speed camera to record the entire process of LM collision and especially the thickness of the pancake shape at a maximum deformation. As plotted in Figure 4b, the curve shows that the thickness of the pancake at the maximum deformation decreases with the increase of the falling height. When the height was larger than 5 cm, the LMs would collapse and the thickness of the pancake is recorded as 0. When the height was in the range of about 3–5 cm (safety height without high‐adhesion wetting for the LM‐substrate impact), the coalescence of LMs would occur efficiently. LM with a height smaller than 3 cm was insufficient to make a coalescence. Consequently, the height of the falling should be within a certain range to obtain an efficient coalescence effect. The impact forces between the LMs at each falling height were estimated at each height as plotted in Figure 4c.

The coalescence process could also be realized by a sliding impact process as is shown in Figure 4e. As proposed in Figure 4b, the LMs collapsed while the falling height is 8 cm. Here, the 6 µL LM was released in the upper terminal of a fixed and tilted hollow pipe with a length of 8 cm and variable tilt angles to trigger coalescence. From the successive side‐view images in Figure S6 in the Supporting Information, when the tilt angle of the hollow pipe is smaller than 30°, the coalesce does not occur and the LMs slide away separately. When the tilt angle of the hollow pipe increased to 60° (Figure 4e), the two LMs could coalesce to form a larger LM intact. The coalescence process completed within a short time of 20 ms. We conclude that the LMs bounced away while the tilt angle was too small, coalesced while the tilt angle was within a certain range, and collapsed while the tilt angle was too large.

Fresh medium and drug additions, as well as coculture of different types of cells, could be achieved by the coalescence of LMs (Figure 4f). The direct addition of medium/drugs through injections with a needle also mixes the bioreactant. However, the manual method is difficult for microliter droplets. More importantly, the ultimate capacity of the LM is limited by the outer hydrophobic particles. The shell only tolerates slight expansion from the addition of an external solution; otherwise, surface defects are generated and the shell easily collapses. The amount of medium injected with a needle is therefore extremely limited. Regarding the fusion‐based mixture, large amounts of medium/bioreactant can be mixed into the original LM since the hydrophobic powder on the two LMs is sufficient to encompass the fused marble without any surface defects.

## 5 Splitting

**Figure 5:**
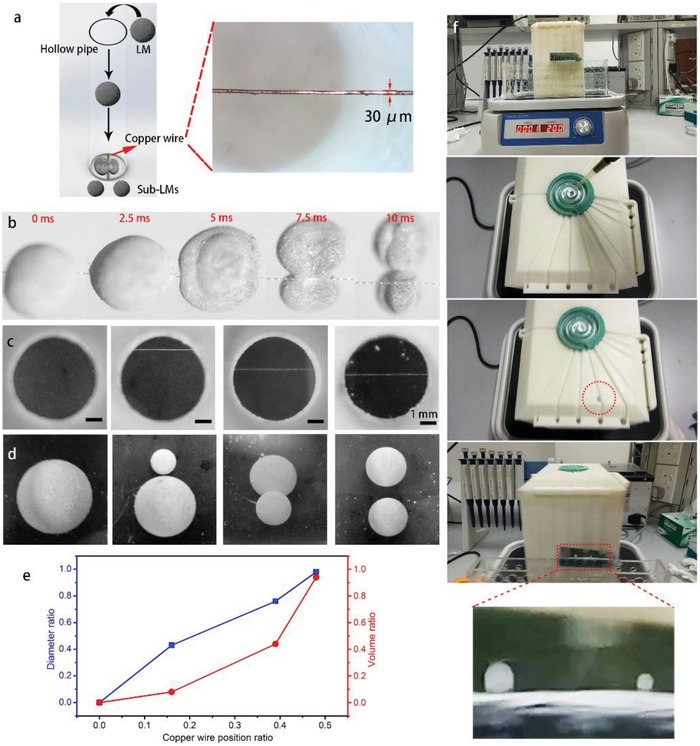
a) Schematic illustration of the splitting processes of LMs. The inset shows the microscopic image of the copper wire with a diameter of 30 µm. b) Successive images showing the top view of a 12 µL LM cut through the center by a copper wire via falling down process at a 2 cm height. The LM was cut into two sub‐LMs (also see Movie S3 in the Supporting Information). c) Top view of the channels (inner diameter is 5 mm) with the copper wires fixed at a different position. d) Optical images showing the LMs separated by the channels assembled with copper wires. e) The relationships between the diameter ratio and the volume ratio of the separated LMs, and the copper wire PR. f) Photographs demonstrating the splitting process of the LM with the CSS.

Apart from the coalescence of two small LMs into a larger one, the inverse process (**Figure** **5**a), which separates one LM into two or several smaller ones can also be realized. Previous work has demonstrated that a manual cutting process could be applied for the splitting of LMs.^45^ Here the splitting of LM could be realized by the simple impact process as is shown in Figure 5. A copper wire with a diameter of 30 µm was tightly straightened and the 12 µL LM was made to fall down from a 2 cm height to impact the copper wire through the sphere center. As shown in Figure 5b, the impact between LM and the copper wire made a severe deformation of the LM and the LM was cut into two counterparts within 10 ms. The surface particles self‐rearranged on the separated two droplet surface during the short impact process, to form evenly distributed sub‐LMs. The size of the sub‐LMs could be controlled by controlling the cutting position of the parent‐LM. As shown in Figure 5c,d, the copper wire was straightened and fixed on the outlet of the channels (diameter is 5 mm) with different positions. The position ratio (PR) represents the value of the shorter part of channel diameter cut by the copper wire divided by the diameter (0 ≤ PR ≤ 0.5). With the increase of the PR, both the diameter ratio and the volume ratio of the obtained sub‐LMs increased (Figure 5e). The larger diameter ratio and volume ratio denote the closeness of the sizes of the sub‐LMs. When the copper wire is fixed across the center of the cross section of the channel, the dropped LM (60 µL) could be cut into two counterparts with 30 µL.

Waste medium removal was achieved by separating the formed LMs using a gravity‐driven cutting process after certain culture period (Figure 5f). After the 3D cell spheroid is formed inside the LM, the gravity‐driven cutting of the LM will separate the LM into two counterparts and the 3D cell spheroid will remain in one of the sub‐LMs. Bright‐field (BF) and fluorescence microscopy are used to screen the LMs by inspecting which LM contained the spheroid.

## 6 3D Culture of Stem Cell Spheroids

The application of LM as a chemical reactor has been reported previously and here we demonstrated this application in Figure S7 in the Supporting Information. The aqueous solution‐based reactions with mild reaction condition can be precisely performed by our coalescence process to form nanomaterials. For example, the synthesis of the Ag NPs was proposed in the LM via an impact‐induced coalescence process and could be easily extracted for further usage. The specific information was presented in Figure S7 in the Supporting Information. Another application we must emphasize is the LM‐based SCS culture by applying our technique.

**Figure 6:**
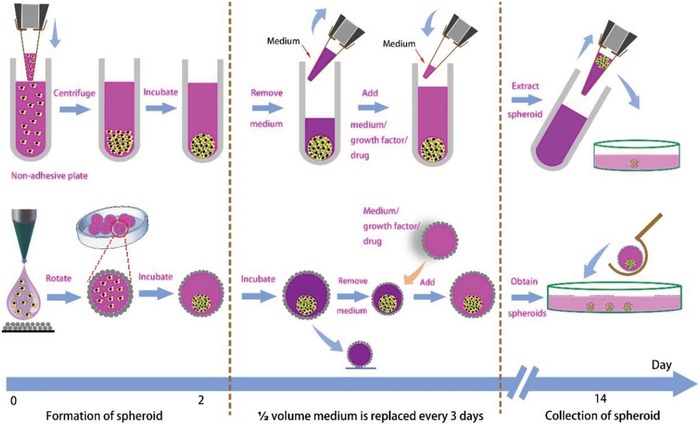
Comparison between the protocols for culturing 3D SCSs using a nonadhesive plate and our strategy.

In a conventional 3D cell culture protocol, cell spheroids are commonly obtained with either nonadhesive plates or the hanging drop method. For nonadhesive plates, the flow of the process includes the addition of culture medium containing stem cells, centrifugation of the medium to form aggregates at bottom of the well, incubation of the stem cells to form spheroids, replenishment of the medium with a pipette, and collection of the spheroids by pipetting using wide bore pipette tips, as shown in **Figure** **6** and Figure S8 in the Supporting Information. The hanging drop method uses similar culture protocols, and compared with the nonadhesive plate method, the hanging droplet is less extendable and the medium of the hanging droplet is difficult to replenish without affecting the inner spheroid. Using our protocol, 3D SCSs were easily harvested with the CSS using the LM as the carrier. As shown in Figure 6, we compared the 3D stem cell culture process of our protocol and the standard nonadhesive plate in three stages divided by the dotted lines. The formation of the spheroid in our protocol applied the hydrophobic silica particles to wrap the medium drop to ensure that the medium was well separated from the outer environment and permitted gas exchange. We applied a splitting process to remove the spent medium and a fusion process to replenish fresh medium. The last stage is the extraction of the LM. Instead of removing the LM from the nonadhesive plate‐based culture with a pipette, the LM was directly poured into the collection container with medium/phosphate‐buffered saline (PBS) solution, followed with a slight shaking process to break the LM. The SCS would then sink to the bottom.

**Figure 7:**
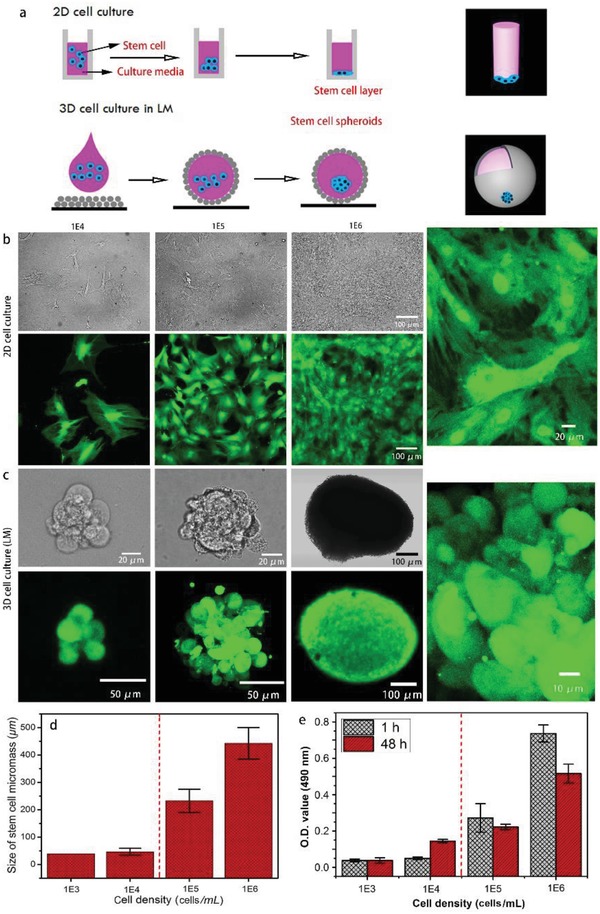
a) Schematic of the strategies for culturing cells in a 2D format and using our method. b,c) FIs showing stem cells cultured on a 96‐well plate b) and in 50 µL of LM c) for 48 h with different initial concentrations of stem cells, i.e., 10^4^, 10^5^, and 10^6^ cells mL^−1^. d) The column chart showing the size of the spheroid at different initial concentrations of stem cells. e) MTS assay showing the O.D. values of stem cells cultured in LMs after short‐term (1 h) and long‐term incubations (48 h). The error bars in (d) and (e) were obtained from 3 to 5 groups of experiments.

We compared the cellular configurations of the standard 2D cell culture and our method at different cell concentrations (**Figure** **7**a), as shown in Figure 7b,c. Stem cells cultured within a standard 96‐well plate spread and formed 2D stem cell layers on the bottom of the plate at various stem cell densities after 48 h in culture. The enlarged fluorescent image (FI) shows the elongated laminar morphology of cells from 2D cell cultures and extensive contacts in the surrounding area. Meanwhile, when stem cells were cultured in 50 µL of LMs for 48 h, cells in the LMs tended to aggregate, forming a large entity known as a 3D spheroid. The enlarged view of the spheroid shows the sphere‐like surface of the spheroid and cells that were tightly bound to one another, as shown in Figure 7c. The size of the SCS increased with the cell density (Figure 7d). As illustrated in Figure 7d, when the cell density was less than 10^4^ cells mL^−1^, spheroids were rarely formed and the size of the cell aggregates was approximately the size of single stem cell. When the cell density was greater than 10^5^ cells mL^−1^, the 3D SCS was well formed, as shown in Figure 7c. Cells cultured in the LMs tended to form single spheroids and the average sizes of spheroids were 250 µm at a cell density of 10^5^ cells mL^−1^ and 450 µm at a cell density of 10^6^ cells mL^−1^ (Figure 7d; Figure S9, Supporting Information). The 3D SCSs that self‐assembled at a density of 10^6^ cells mL^−1^ was tightly packed with ≈5000 cells in each spheroid, creating an “in vivo‐like” micro‐bioenvironment for organoid development that better preserved the stem cell phenotype and its innate properties. In a recent publication, the researchers suggested that the liquid marble–based 3D stem cell culture showed its potential applications in embryonic body formation and differentiation.^46^

The viability of cells growing inside the LMs was determined using the 3‐(4, 5‐dimethylthiazol‐2‐yl)‐5‐(3‐carboxymethoxyphenyl)‐2‐(4‐sulfophenyl)‐2H‐tetrazolium (MTS) assay. As shown in Figure 7e, the optical density (O.D.) at 490 nm was measured for stem cells cultured in LMs for 1 and 48 h. The cell viability of the 1 and 48 h cultures were comparable when the cell density was less than 10^4^ cells mL^−1^, which may be attributed to the lack of spheroid formation at low cell concentrations. Cells were uniformly dispersed inside the LMs and had a sufficient number of contacts with the culture medium. When the initial cell density was greater than 10^5^ cells mL^−1^, the cell viability of the 48 h culture was slightly lower than the 1 h culture, since the stem cells in the 48 h culture formed tightly bonded spheroids. As proposed in a previous report,^47^ cell spheroids exhibited three zones along the radius, i.e., a proliferating zone, quiescent viable cell zone, and necrotic core. The spherical cell aggregates represent an avascular tissue with a limit of diffusion of ≈150–200 µm for many molecules, including O_2_.^48^ Since molecules are mainly digested in the proliferating zone and the cell viability in the 48 h culture was less than the 1 h culture, a well‐formed SCS developed when the cell density was greater than 10^5^ cells mL^−1^.

In addition to the apparent cells, the internal cell‐to‐cell communication in the spheroid was inspected by capturing images with a confocal laser scanning microscope (CLSM) layer‐by‐layer with a gap of 9 µm, as shown in Figure S10 in the Supporting Information. Cells inside the spheroid (derived from a cell density of 10^5^ cells mL^−1^) also showed a compact configuration. The shape of the cells varied, enabling them to conform to the environment. The cells inside the spheroid were in different shapes. The shape diversity inside a spheroid can affect the differentiation direction of stem cells. For instance, the chondrogenesis requires the stem cell to be spherical shape whereas the osteogenesis requires the stem cell to be spindle shape.

The in situ inspection of the spheroid in an LM with BF and fluorescence microscopy is shown in Figure S11 in the Supporting Information. After the cutting‐based splitting, the LM containing spheroid was then used in another coalescence process with an LM containing fresh medium using our system and the other LM was subjected to further analysis and disposals. We can quickly inspect whether the separated LMs contain an SCS with the BF and fluorescent microscopy without breaking the LM (Figure S11, Supporting Information).

**Figure 8:**
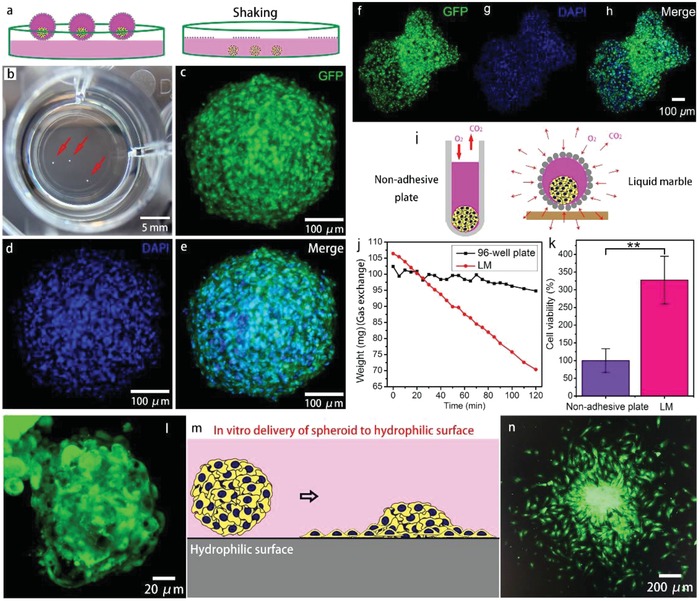
a) Schematic of the batch extraction process used for the SCSs in LMs. b) The 3D SCSs cultured in LMs at a cell density of 10^6^ cells mL^−1^ were visible to the naked eye. The 3D SCSs cultured in the LMs were released into the PBS solution by simply shaking the plate with the LMs on the surface of the PBS solution. The 3D SCSs visible to the naked eye sank and were easily extracted with a pipette/dropper. c–e) CLSM images of the 3D spheroids cultured in LMs at a cell density of 10^6^ cells mL^−1^. FITC and DAPI channels were examined. f–h) CLSM images of a 3D spheroid cultured in a 96‐well nonadhesive plate at a cell density of 10^6^ cells mL^−1^. i) Gas exchange in the two types of culture. j) The evaporation curve of 100 µL of the culture medium in 96‐well plate and LM at room temperature in an open environment. k) The results of the MTS assay showing the viability of stem cells cultured at a cell density of 10^6^ cells mL^−1^ in 96‐well nonadhesive plates (control) and LMs. The error bars shown in (j) were obtained from 3 groups of experiments. Asterisks denote the level of significance: ** *p* < 0.01. l,n) The CLSM images of the morphology of a SCS with two days' culture period in the LM and the morphology after delivery onto a hydrophilic surface for another two days' culture. m) Schematic of the change of the cell morphology after delivery and replanting process (in vitro).

The SCS derived from our system at a cell density of 10^6^ cells mL^−1^ was visible to the naked eye after the LM on medium/PBS solution was subjected to a simple shaking process, as indicated by the arrows in **Figure** **8**a,b; this step is important and quite easy for the handling of the spheroid for further applications. We compared the cell configurations in the nonadhesive plate‐based 3D cell culture with our method at the same cell concentration, as shown in Figure 8. The 2D control group cultured in a 96‐well plate is shown in Figure S12 in the Supporting Information. Cells emitted green fluorescence under blue light illumination due to the expression of the green fluorescent protein (GFP), and the cell nuclei were stained with 4′,6‐diamidino‐2‐phenylindole (DAPI) to label the nuclei with blue fluorescence. Compared with the spheroids obtained from standard 3D cell culture with nonadhesive plates, the cell configurations resulting from the nonadhesive plate and LM indicated that the sizes of the spheroids were comparable, with some differences in shape, as shown in Figure 8c–h. The viability of the cells in the spheroids cultured using these two methods was evaluated with the MTS assay by measuring the O.D. value at 490 nm, as shown in Figure 8k. The viability of cells cultured in the spheroid using the LM was approximately threefold higher than cells cultured on the nonadhesive plate, denoting the better cell viability of the LM‐based spheroid. The high cell viability of the spheroid produced using our method may be attributed to the structural advantage of the LM compared with the standard nonadhesive plate. As CO_2_ and O_2_ are indispensable for the cell metabolism, the LM is gas‐permeable and contains numerous microscale pores on the LM surface (Figure S13, Supporting Information) that enable the medium inside the LM to participate in gas exchange with the surrounding environment, whereas the standard nonadhesive plate only participates in gas exchange on the upper surface of the medium, as schematically illustrated in Figure 8i. We measured the weight variation of 100 µL of culture medium in a 96‐well plate and 100 µL of culture medium in an LM over time at room temperature in an open environment to confirm that gas exchange was more efficient in the LM. As shown in Figure 8j, greater changes in weight were observed for the LM than the 96‐well plate (6.5‐fold), indicating better gas exchange by the stem cells in the LM.

The obtained 3D spheroid with high‐viability may show their potential for regenerative medicine, such as tissue repairing. As demonstrated in Figure 8m, after two days' culture in LM, the cells form a spheroid, the spheroid can be directly delivered and planted onto a hydrophilic surface with ease for the spreading and diffusing. Figure 8l,n gives the CLSM images of the morphology of an SCS with two days' culture period in the LM and the morphology after delivery onto a hydrophilic surface for another two days' culture. The result also suggests that the morphology of the individual stem cells changed from the sphere to the fried egg shape with an increased cell‐substrate contact area. Meanwhile, the results further proved the high cell viability of the spheroid.

The comparison of 3D cell culture tools is described in detail in **Table** **1**, showing evaluations of the size and shape of the formed spheroid, the cell viability, the internal cell‐to‐cell communication of the as‐obtained spheroid, the complexity of the extraction of the as‐cultured spheroid, the manipulation process, and the toxicity of the materials. As illustrated in the table, a standard culture dish is commonly used for 2D spread cell cultures and cell spheroids rarely develop. The advantages of our CSS include the formation of a viable spheroid with a wide range of sizes using simple manipulation strategy compared with the other tools. Nonadhesive plates are limited by their ability to obtain spheroids at high cell viability. Hanging drop and spinner flask cultures are limited by the ability to culture spheroids with large sizes or controllable shapes. In addition, the hanging drop method, 3D scaffolds, and magnetic cell levitation‐based cultures introduce external materials that have close contact with the cells and may produce long‐term toxicity to the cells. Overall, our CSS offers researchers a choice for producing SCSs with a large range of size, high cell viability, strong internal cell‐to‐cell contact, a simple extraction process, and low toxicity of the materials.

**Table 1:** Comparison of 3D cell culture methods

Method	Spheroid size	Spheroid shape	Cell viability	Cell‐to‐cell contact	Extraction of spheroid	Process	Materials toxicity
Standard culture dish^49^	n.a.	n.a.	+++	Only at edge (2D)	n.a.	Easy and standard	None
Nonadhesive plate^50^	Large (≈100–1000 µm)	Spherical	+	Sufficient in 3D	Simple	Easy and standard	None
Hanging drop^51^	Generally <500 µm	Spherical	++	Sufficient in 3D	Simple	Easy and standard	None
3D scaffolds^52^	Large and tunable (Up to macroscale depend on the size of scaffolds)	Controllable	++	Mainly contact with scaffold	Simple	Sophisticated to fabricate the scaffold	Potential toxicity of the scaffold
Spinner flask^49^	Large amount with small size (microscale)	Irregular	++	Sufficient in 3D	Simple	Simple spinning process	None
Magnetic cell levitation^53^	Large (<1 mm)	Quasi‐spherical	++	Cell‐to‐cell and cell‐to‐particles contact both exist	Simple	Additional magnetic levitation step based on culture dish	Potential toxicity of the nanomaterials
Microfluid^54^	Generally <500 µm	Spherical	+	Sufficient in 3D	Difficult due to the oil–water barrier	Easy and automated for droplet generation	Low‐toxicity
CSS	Large (≈100–1000 µm)	Spherical	++	Sufficient in 3D	Simple shaking of LM on PBS solution	Relatively simple due to the manipulability of LMs	Low‐toxicity

## 7 Conclusion

In summary, the coalescence and splitting of LMs by applying simple mechanical force were systematically investigated to obtain the optimal parameters for the processes. By selecting the functional channels on the device, gravity‐based fusion and splitting of LMs were performed to mix precise volume of medium/drugs and remove spent culture medium, thus ensuring that the microenvironment of the cells was maintained under optimal conditions. The size of the cell spheroid was controlled within a wide range, up to sub‐millimeter in size. The cultured SCS showed highly compact cell‐to‐cell cross‐contacts, which are more valuable for the simulation of a real in vivo tissue than the 2D cell monolayer. The high gas permeability and the liquid repellent properties of the formed LMs endow the “in vitro tissue” with good cell viability, which is crucial for stem cell differentiation and tissue engineering. After transferring and delivering the cell spheroid to a hydrophilic surface, the cells were respread and transformed into typical 2D cell morphology gradually, which may offer the possibility for spheroid‐based stem cell delivery. The system offers researchers a new choice for producing SCSs with a large size, high cell viability, and strong internal cell‐to‐cell contact, which is a useful in vitro model to study the formation and growth mechanism of tissues in vivo. The coalescence and splitting of LMs show the great prospect for ultrafast, tiny, and stoichiometric chemical reactions, synthesis of functional nanomaterials, and cell spheroid culture in biomedical engineering.

## Conflict of Interest

The authors declare no conflict of interest.

## Supporting information

Supplementary

Supplementary

Supplementary

Supplementary

Supplementary
