# Supplementary material for: On‐Demand Coalescence and Splitting of Liquid Marbles and Their Bioapplications
Source: Adv Sci (Weinh). 2019 Mar 14;6(10):1802033. doi: 10.1002/advs.201802033 (PMC6523389; doi:10.1002/advs.201802033)
Supplement: Supplementary file 1 — Supplementary [file ADVS-6-1802033-s002.pdf]

Copyright WILEY-VCH Verlag GmbH & Co. KGaA, 69469 Weinheim, Germany, 2019.

ADVANCED SCIENCE
Open Access

Supporting Information

for *Adv. Sci.,* DOI: 10.1002/advs.201802033

On-Demand Coalescence and Splitting of Liquid Marbles and Their Bioapplications

Ben Wang, Kai Fung Chan, Fengtong Ji, Qianqian Wang, Philip Wai Yan Chiu, Zhiguang Guo, and Li Zhang**

Supporting Information

On-demand coalescence and separation of liquid marbles and their bio-applications

Ben Wang†[a,b], *Kai Fung Chan*†[b,d], Fengtong Ji[a], Qianqian Wang[a], Philip Wai Yan Chiu[d,e], Zhiguang Guo*[c], Li Zhang*[a,d,e]

[a]Department of Mechanical and Automation Engineering, The Chinese University of Hong Kong, Hong Kong, China

[b]Department of Biomedical Engineering, The Chinese University of Hong Kong, Hong Kong, China

[c]State Key Laboratory of Solid Lubrication, Lanzhou Institute of Chemical Physics, Chinese Academy of Sciences, Lanzhou 730000, China

[d]Chow Yuk Ho Technology Centre for Innovative Medicine, The Chinese University of Hong Kong, Hong Kong, China

[e]Department of Surgery, The Chinese University of Hong Kong, Hong Kong, China

[f]T Stone Robotics Institute, The Chinese University of Hong Kong, Hong Kong, China

† B.W. and K.F.C contributed equally to this work.

Correspondence and requests for materials should be addressed to Z.G. (zguo@licp.cas.cn) and

L.Z. (lizhang@mae.cuhk.edu.hk)

Experimental Section

Materials: Ammonium hydroxide, trimethoxymethylsilane, and resazurin were obtained from Aladdin Chemical Co., Ltd (Shanghai, China). Dulbecco's Modified Eagle's Medium (DMEM), fetal bovine serum (FBS), 0.25% trypsin-ethylenediaminetetraacetic acid (EDTA), 4′,6-diamidino-2-phenylindole (DAPI), phosphate-buffered saline (PBS) and Nunclon Sphera 96U-well plates were purchased from GIBCO-Thermo Fisher Scientific. The 3-(4, 5-dimethylthiazol-2-yl)-5-(3-carboxymethoxyphenyl)-2-(4-sulfophenyl)-2H-tetrazolium (MTS) assay (ab197010) was obtained from Abcam. The 4% paraformaldehyde solution and Triton X-100 were purchased from Sigma-Aldrich (USA). All chemicals were used as received without further purification.

Preparation of hydrophobic SiO_2 microspheres: The SiO_2 microspheres were prepared using the method reported by Stöber[1], with some modifications. Briefly, 3 mL of ammonium hydroxide (28% wt.) were poured into a mixture of 150 mL of ethanol and 15 mL of water in a flask with magnetic stirring. Then, 9 mL of TEOS were added to the above mixture in a dropwise manner. The reactant was vigorously stirred for 3 h. Next, the white precipitate was collected by centrifugation and washed with deionized water three times. The final white powder was obtained by drying the precipitate in a drying oven at 60°C overnight. The white powder was then immersed in 20 μL n-hexane per 1 mL of trimethoxymethylsilane for 12 h at room temperature. The precipitate was then washed with n-hexane and ethanol several times and dried in a drying oven.

Liquid marble formation: The dried hydrophobic SiO_2 powder was poured onto the 3D-printed spiral dish. LMs were obtained by simply dropping a liquid droplet on the center of the dish and agitation on the orbital shaker.

ADMSC culture in liquid marbles: Adipose-derived mesenchymal stem cells (ADMSCs) were isolated from subcutaneous inguinal adipose tissue of male transgenic Sprague-Dawley (SD) rats expressing green fluorescent protein (GFP). ADMSCs were used at passages 2–5 in the subsequent experiments. Adherent ADMSCs were first grown to 80 - 90% confluence. They were then detached by incubating them with a 0.25% trypsin-EDTA solution for 50 s at 37 °C. Once the

cells had detached from the dishes, trypsinization was stopped by immediately adding a double volume of DMEM supplemented with 10% FBS. The cell suspension was centrifuged at 1000 rpm for 5 min. The cell pellet was resuspended in 1 ml of DMEM containing 10% FBS. The cell density was determined using a hemacytometer. ADMSC stocks at different cell densities (1E3, 1E4, 1E5 and 1E6 cells/ml) were prepared. Afterward, 50 μL of ADMSC stock solutions of different cell densities were dropped onto the SiO_2 powder on the culture dish. The SiO_2 powder was pre-sterilized in an autoclave. The LMs containing ADMSCs were obtained by rolling a medium droplet on the bed of the SiO_2 powder. The resulting LMs were then transferred to a sterile spoon, and the spoon was gently tapped to remove the loosely attached nanoparticles on the LMs. LMs were then transferred to the central wells of a 96-well plates. Empty wells were filled with sterile phosphate-buffered saline to reduce the evaporation of the LMs. The LM culture was incubated at 37 °C in a 5% CO_2 incubator for different periods of time.

Cell viability assay in liquid marble cultures: The viability of ADMSCs cultured in LMs was measured using the MTS assay. Briefly, ADMSCs were cultured in LMs and Nunclon Sphera 96U-well plates for different periods of time. For LMs, 20 μL of MTS solution and 130 μL of DMEM were added to each LM to promote bursting. For 96U-well plates, 20 μL of MTS solution and 80 μl of DMEM were added to each well. Cells that had originally been cultured using both conditions were then cultured for 3 hours at 37 °C in a 5% CO_2 incubator. Finally, 100 μL of each sample solution were pipetted into a new 96-well plate. The absorbance was detected at 490 nm using a microplate reader. All measurements were repeated at least three times. The cell viability for 3D cell culture applied the non-adhesive 96U-well plates as the reference.

Immunofluorescence staining: ADMSCs at different cell densities (1E3, 1E4, 1E5 and 1E6 cells/ml) were seeded in LMs, Nunclon Sphera 96U-well plates and on glasses placed in a 6-well plate. After 48 hours of culture, ADMSC spheroids in LMs and Nunclon Sphera 96U-well plates were collected and transferred to a new 24-well plate. All cell samples were rinsed three times with PBS and then fixed with 4% paraformaldehyde for 30 minutes at room temperature. Afterwards, the fixed cells were rinsed three times with PBS, followed by permeabilization with 0.5% Triton X-100 for 30 minutes. The permeabilized cells were stained. The nuclei were stained with DAPI diluted with PBS (1:1000) for 30 minutes. After three washes with PBS, fluorescence images were then observed and captured using a Zeiss LSM 700 confocal laser scanning

microscope. All staining procedures were conducted in a dark environment to avoid photobleaching.

Characterization: The contact angles were measured under a DSA25S system (Krüss Company, Germany) by depositing water droplet on the surface the particles. The average contact angle value was obtained by measuring the sample at five different positions. The optical microscopy images were obtained using an HIROX RH-2000 microscope. Scanning electron microscopy (SEM) images of Au-sputtered specimens were captured with a JEOL JSM-7800 scanning electron microscope equipped with an X-ray energy dispersive spectrometer (EDS) attachment. Transmission electron microscopy (TEM) images were captured with a JEOL Model JEM-2011 system equipped with an EDS attachment.

[1] W. Stöber, A. Fink, J. Colloid Interface Sci. 1968, 26, 62-69.

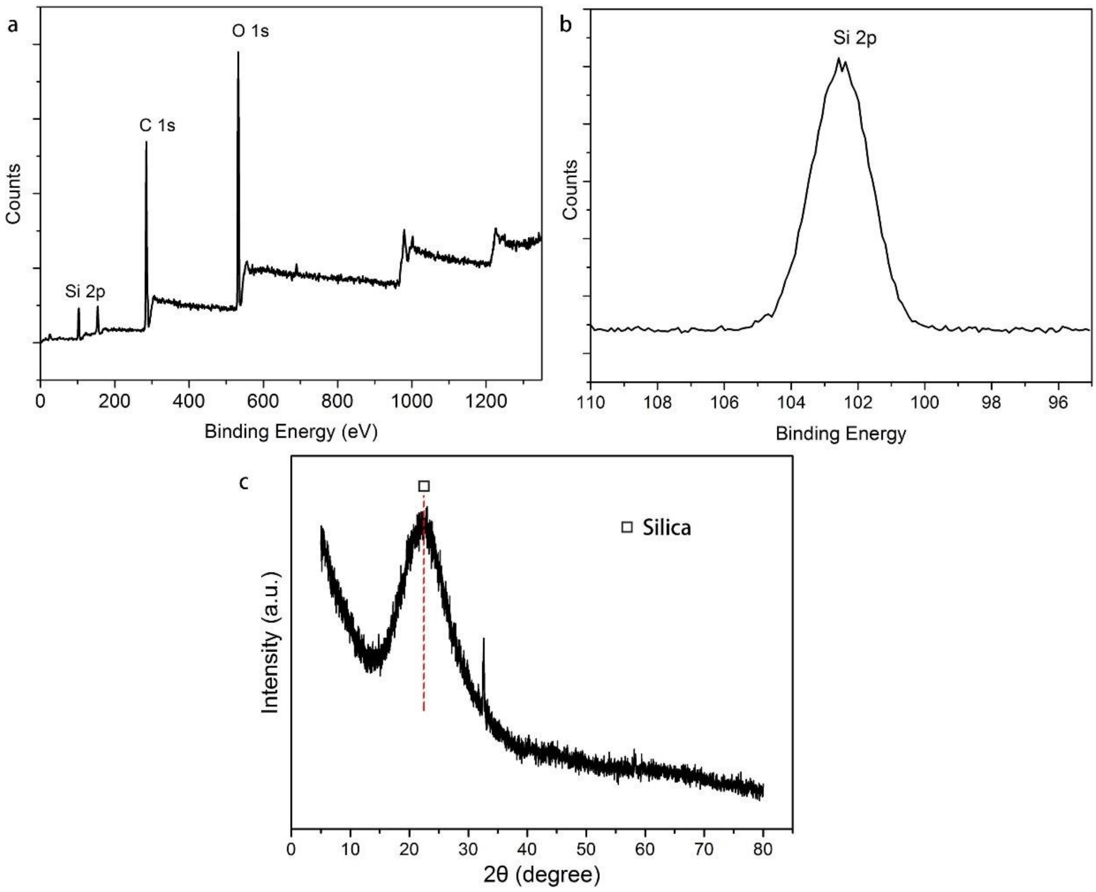

Figure S1 (a and b) XPS spectra of the as-prepared hydrophobic SiO_2 particles. (c) XRD pattern of the as-prepared hydrophobic SiO_2 particles. The chemistry of the particles was investigated by XPS and XRD. The Si 2p peaks in XPS spectrum and the broad peak located at $2\theta = 22.9°$ in XRD diffractogram indicated the powder was silicon dioxide. The characteristic amorphous peak is attributed to silica according to the JCPDS data with card No. 01-086-1561.

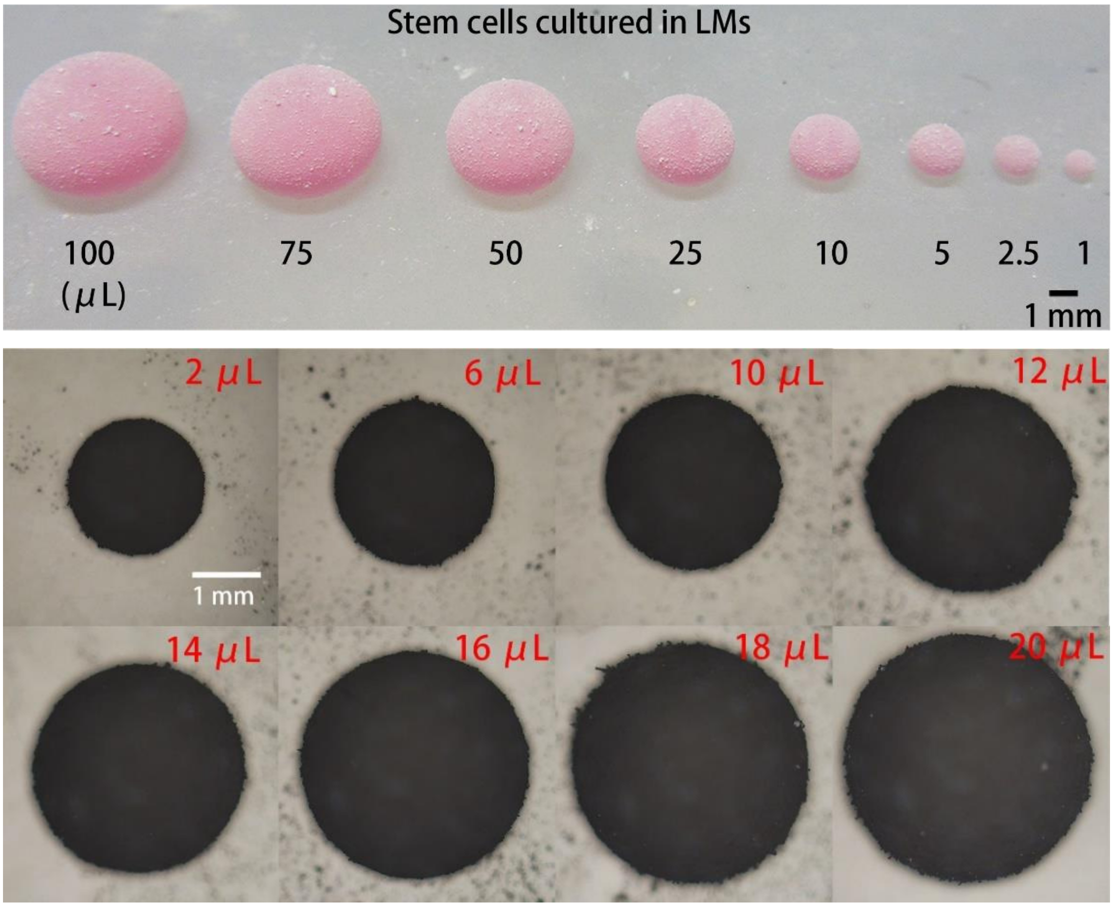

Figure S2 LMs with cultured stem cells inside. The experimental results indicate that LMs have controllable sizes with a wide range of their diameters.

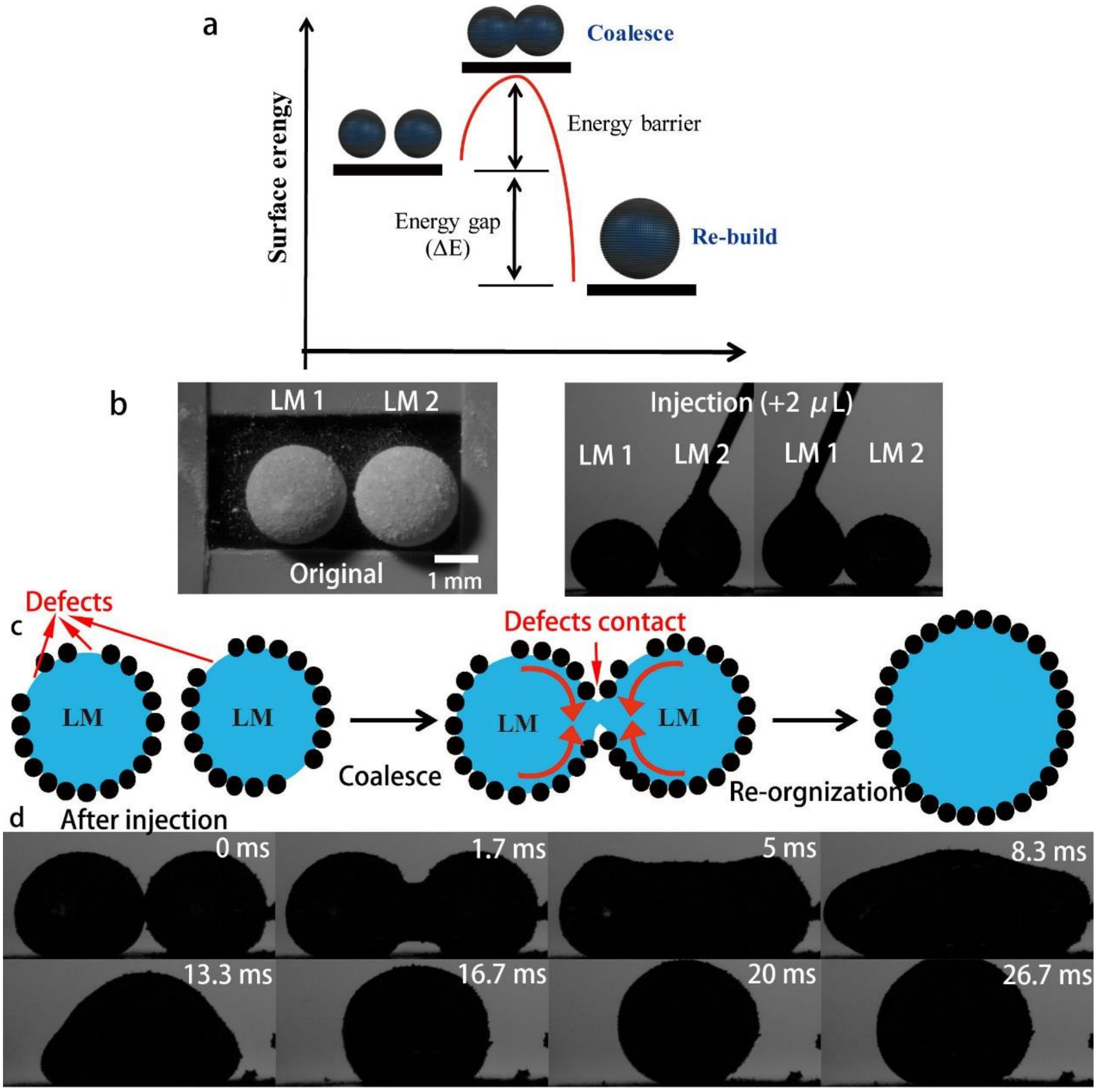

Figure S3 (a) Schematic illustration of the surface free energy variation during the fusion process. The contacts between the LMs are solid-to-solid contact and solid-to-liquid contact. The liquid inside the LMs should separately penetrate through outer hydrophobic powder to touch each other by a liquid-to-liquid contact instead of the solid-to-solid contact. This process required the external energy to overcome the energy barrier. After liquid-to-liquid contact occurred, the fusion of the LMs would be a spontaneous process. (b) Photo images shows the coalescence of the two LMs before coalescence. (c) Schematic illustration of the coalescence process of two LMs with surface defects. (d) Optical images show the coalescence process of two LMs with surface defects.

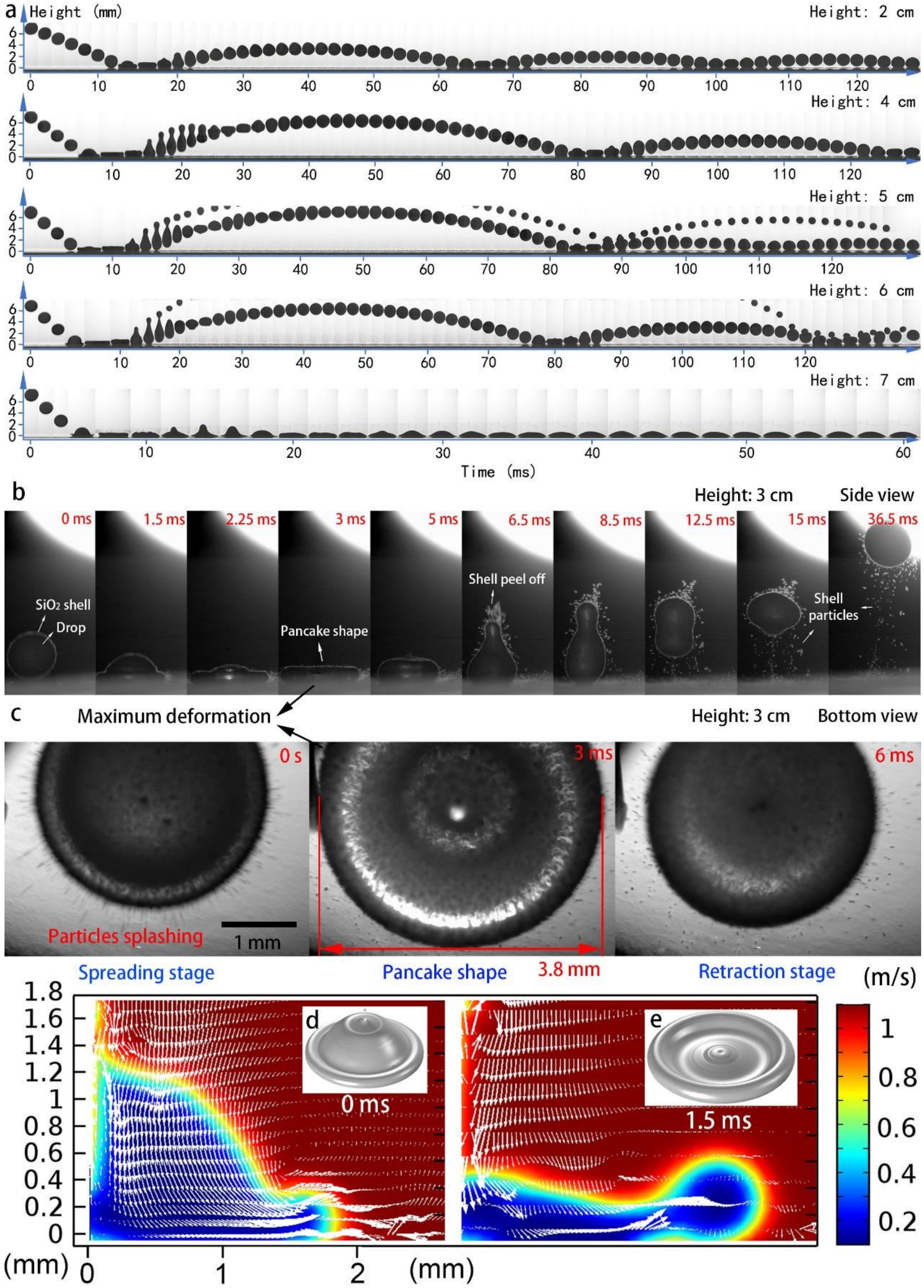

Figure S4. (a) Still frames of speedy movies depicting LM (6 μL) impacting a glass slide with different heights (from 2 ~ 7 cm). (b) Side view of successive optical images showing the enlarged view of the moment of first impact process of LM (6 μL) impact glass slide (3 cm). (c) Bottom view of successive optical images showing the enlarged view of the moment of first impact process of LM (6 μL) impact glass slide (3 cm). (d and e) Simulation results of the collision process of a drop and superhydrophobic surface driven by gravity at 5 cm height via COMSOL.

The vector direction of inner fluid velocity was depicted by the arrows and the length of the arrows is in accordance with the velocity value. Color refer to the value of fluid velocities inside the LM (m/s). (See also Movie S2)

To interpret the coalescence process, we assume that the friction of LM in air is neglectable. For the falling down process of the upper LM, LM falling from a height H is a transformation of the gravitational potential energy to the kinetic energy. According to the theorem of kinetic energy,

$$mgH = 1/2m\upsilon^2$$

where the m represents the mass of the LM and υ represents the velocity before the collision. For the impact process, the impact between the two LMs occurred within a quite short time of t. According to the theorem of momentum,

$$Ft = mv$$

Therefore, the impact force between LMs can be expressed as

$$F = m(2gH)^{1/2}/t$$

Here the weight of the 6 μL LM is measured to be m = 5.4 mg, the impact duration is deemed as the time from initial contact of two LMs to the maximum deformation of the LMs, it's about 6 ms as is shown in Figure 5d. Then we can estimate the impact force between the LMs at each falling height as plotted in Figure 5c. We conclude that the coalescence process of the LMs could occur while the interaction force between LMs is within the range of about 0.7 ~ 0.9 N.

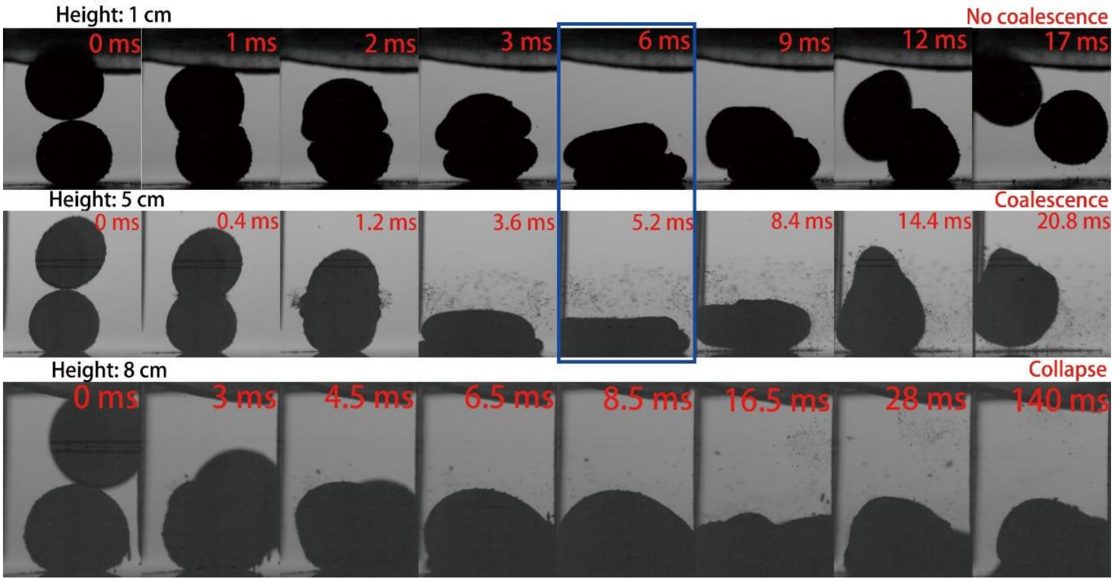

Figure S5 Three groups of sccessive images show the side view of the impact of two LMs at different falling heights (i.e. 1 cm, 5 cm and 8 cm).

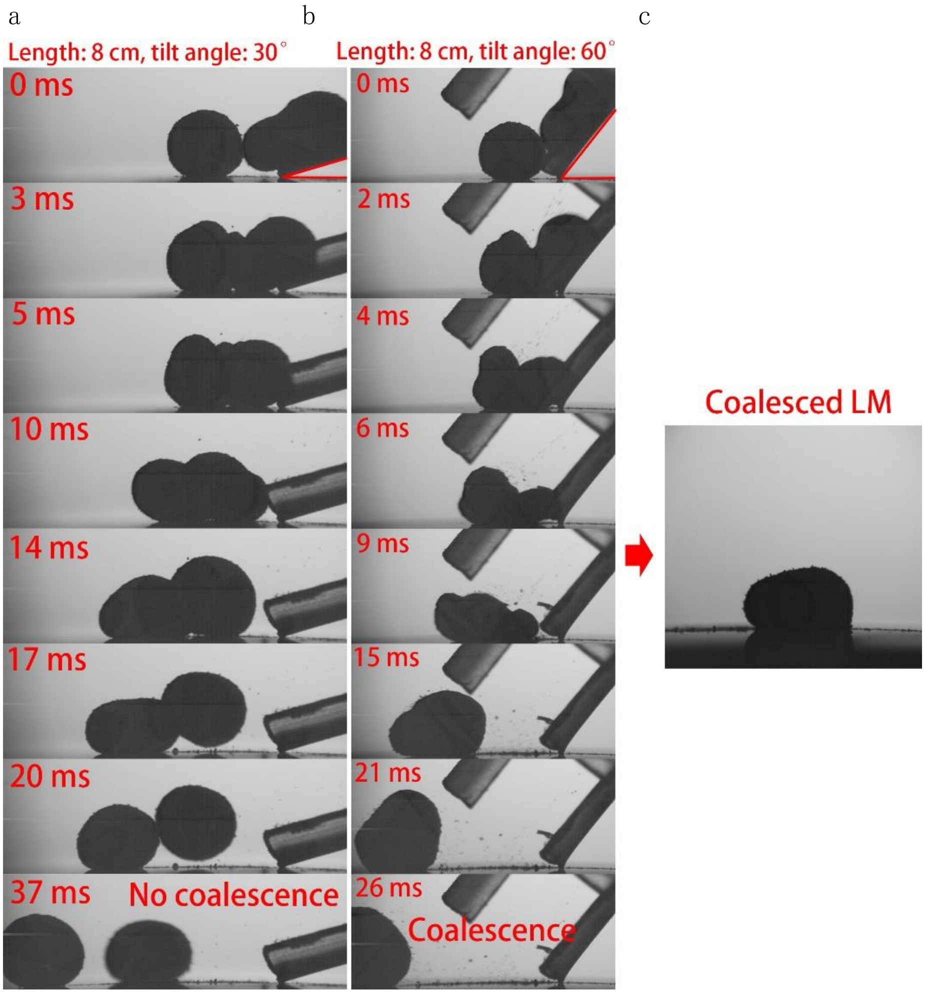

Figure S6 (a) LM impacts LM by falling down a LM from an 8 cm hollow pipe with a tilt angle of 30°. (b) LM impacts LM by falling down a LM from an 8 cm hollow pipe with a tilt angle of 60°. (c) The projected view of the coalesced LM.

As the as-prepared hydrophobic particles could sustain both acid and base solutions and is chemically inert to the inner liquid, many kinds of reactants could be respectively encapsulated into the isolate LMs. The inner liquid droplets could be coalesced in sequence as needed. As an example, the synthesis of the Ag NPs was proposed in the LM via an impact-induced coalescence process. As showed in Figure S6a, the aqueous solution of 10 mM $AgNO_3$ and 1 mM CTAB was prepared and termed as solution A. The aqueous solution of 10 mM glucose and 50 mM NaOH was prepared and termed as solution B. The LM (20 μL) of solution A was fallen from a 3 cm height and hit the 20 μL LM of solution B at the bottom to coalesce into an integral ellipsoid LM as is shown in Figure S6b. The color of the coalesced LM turned from white to black within several second. After several minutes, the inner product was extracted by a pipette (Figure S6c) and collected for the materials characterization (Figure S6d). The SEM images in Figure S6e and f shows the surface morphology of the as-synthesized AgO NPs in the coalesced LM. The AgO NPs show oval geometry with an average particle size of about 30 nm (Fig. S6e and f).

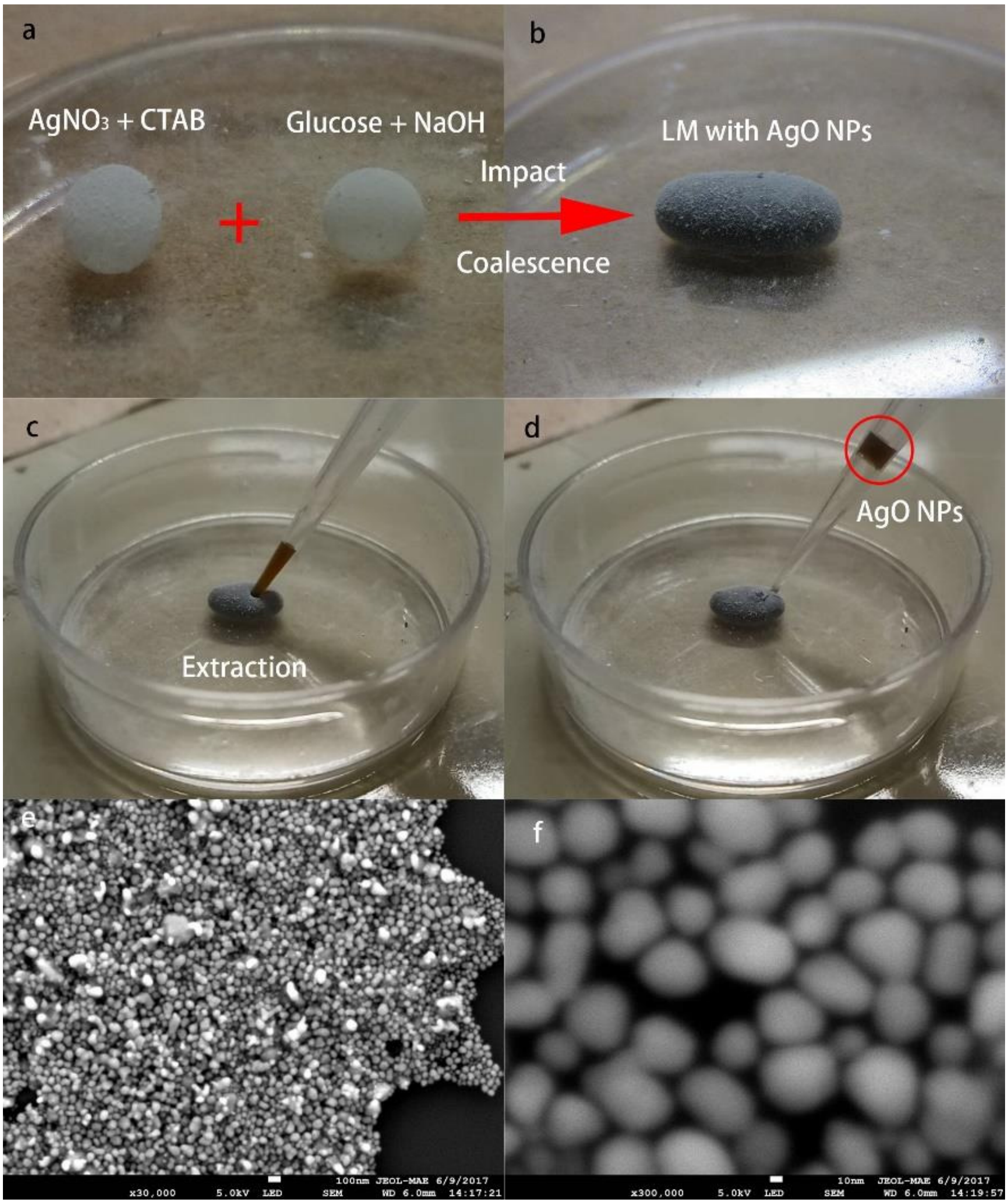

Figure S7 (a) LMs filled with precursors (20 μL). One of the LM is filled with 10 mM $AgNO_3$

and 1 mM CTAB (LM-1), the other one is filled with 10 mM glucose and 50 mM NaOH (LM-2). (b) The LM1 was lift to a height of 3 cm to fall and impact the LM2 to induce coalescence, forming the ellipsoid and black LM. (c) Photo shows the extraction process of the inner product by a pipette. (d) Photo shows the inner product was successfully collected. (e) SEM image shows the integral view of the AgO NPs obtained from the LM micro-reactor. (f) SEM image shows the magnified view of the AgO NPs

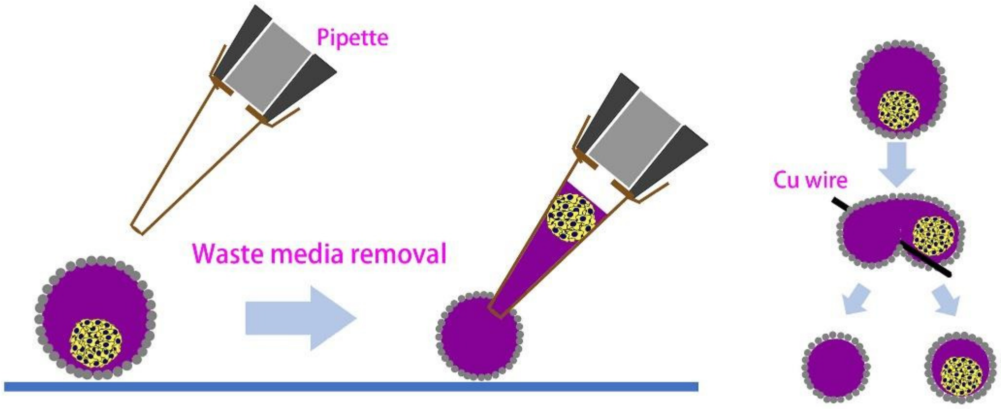

Figure S8 Schematic illustration of extraction process of the waste media using a pipette and our lab-in-a-marble device. Apart from the fusion of two LMs into a larger one, the inverse process, which separate one LM into two or several smaller ones is also demonstrated for purpose of waster media removal after a long-time culturing. The inner waste media of LM can be also extracted with a pipette as shown in Figure S7. However, the method shows a tremendous risk to remove the micromass inside the LM during the process. Therefore, we developed the gravity-driven cutting process to separate one LM into two sub-LMs (Figure S7).

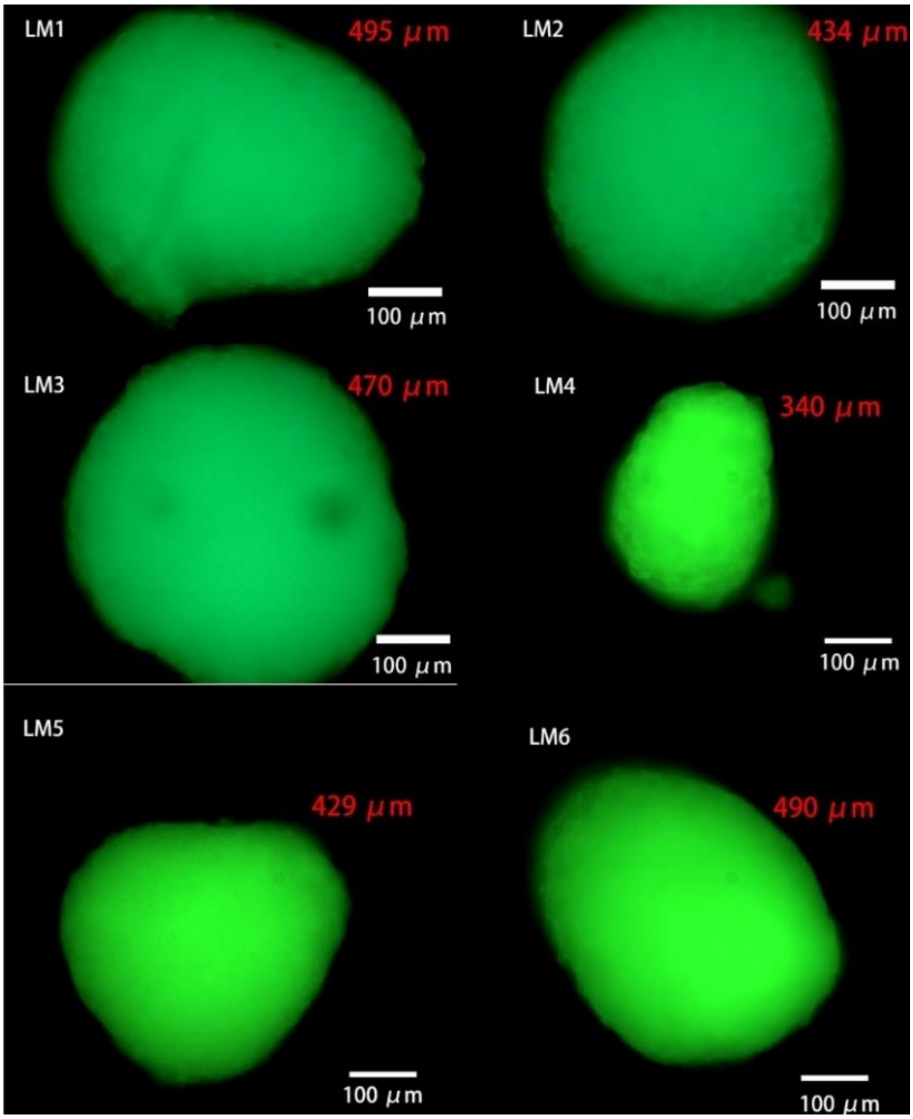

Figure S9 Florescence images show the stem cells spheroids obtained from our protocol with a cell concentration of 1E6 cells/mL.

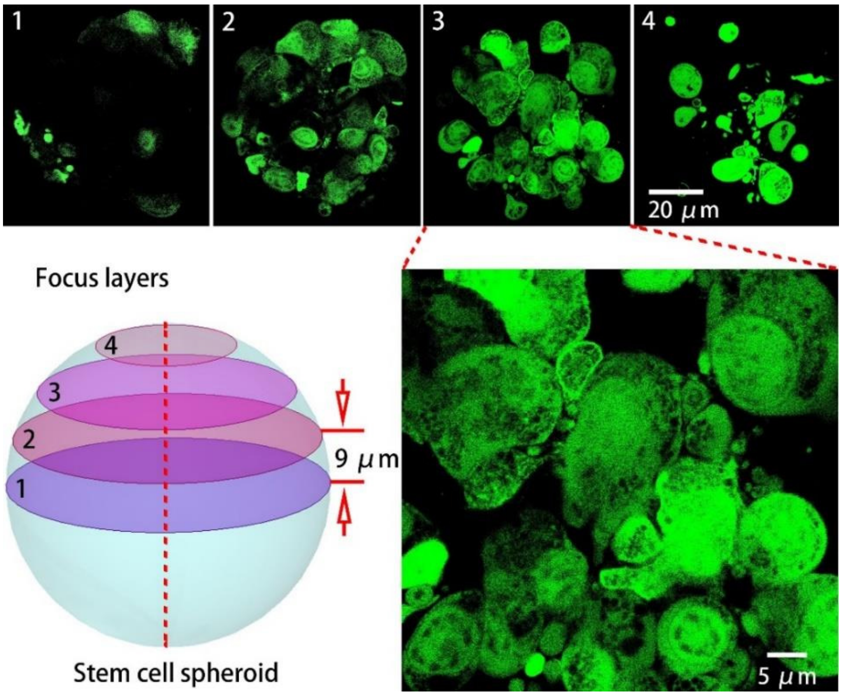

Figure S10 CLSM images show the internal cell-to-cell communication in the spheroid at different layers (derived from a cell density of 1E5 cells/mL).

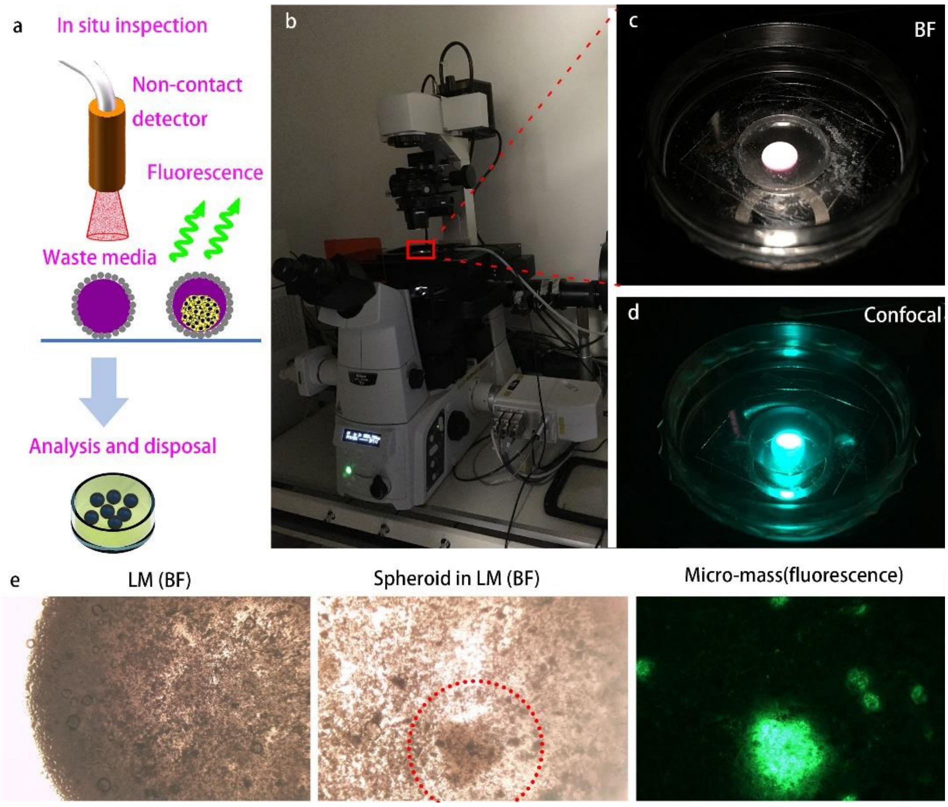

Figure S11. (a) Schematic of the in-situ inspection of the LMs. The process is applied to distinguish the LM of waste media and LM of spheroid after the LM splitting process. (b) Fluorescence microscope setup with the LMs reside on the stage. (c) BF inspection of LM. (d) Fluorescence inspection of LM. (e) BF image of LM and spheroid, and fluorescence image of the spheroid.

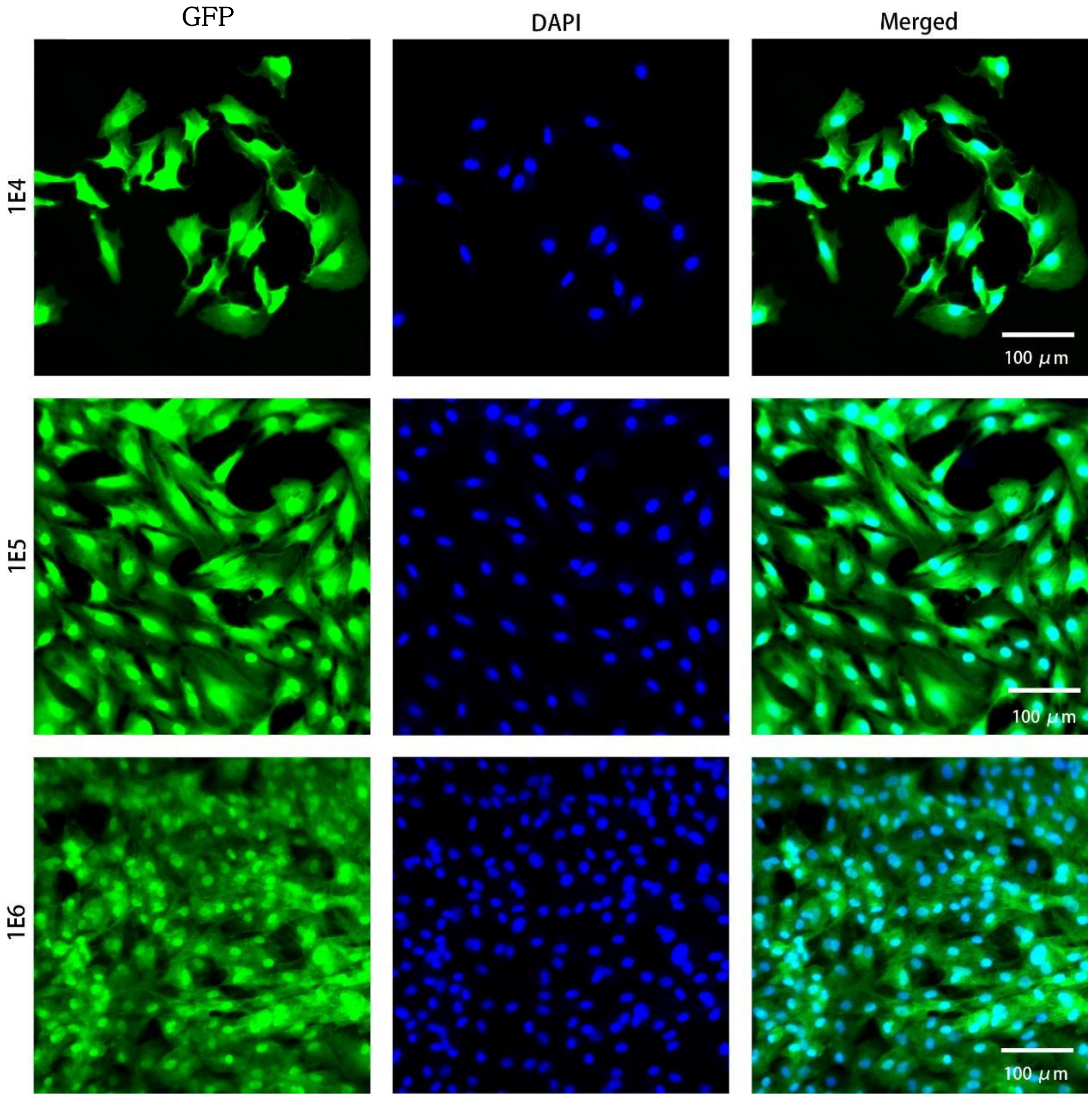

Figure S12 CLSM images of the 2D stem cell cultured in a 96-well plate with cell densities of 1E4, 1E5, and 1E6 cells/mL. The FITC and DAPI channels were examined.

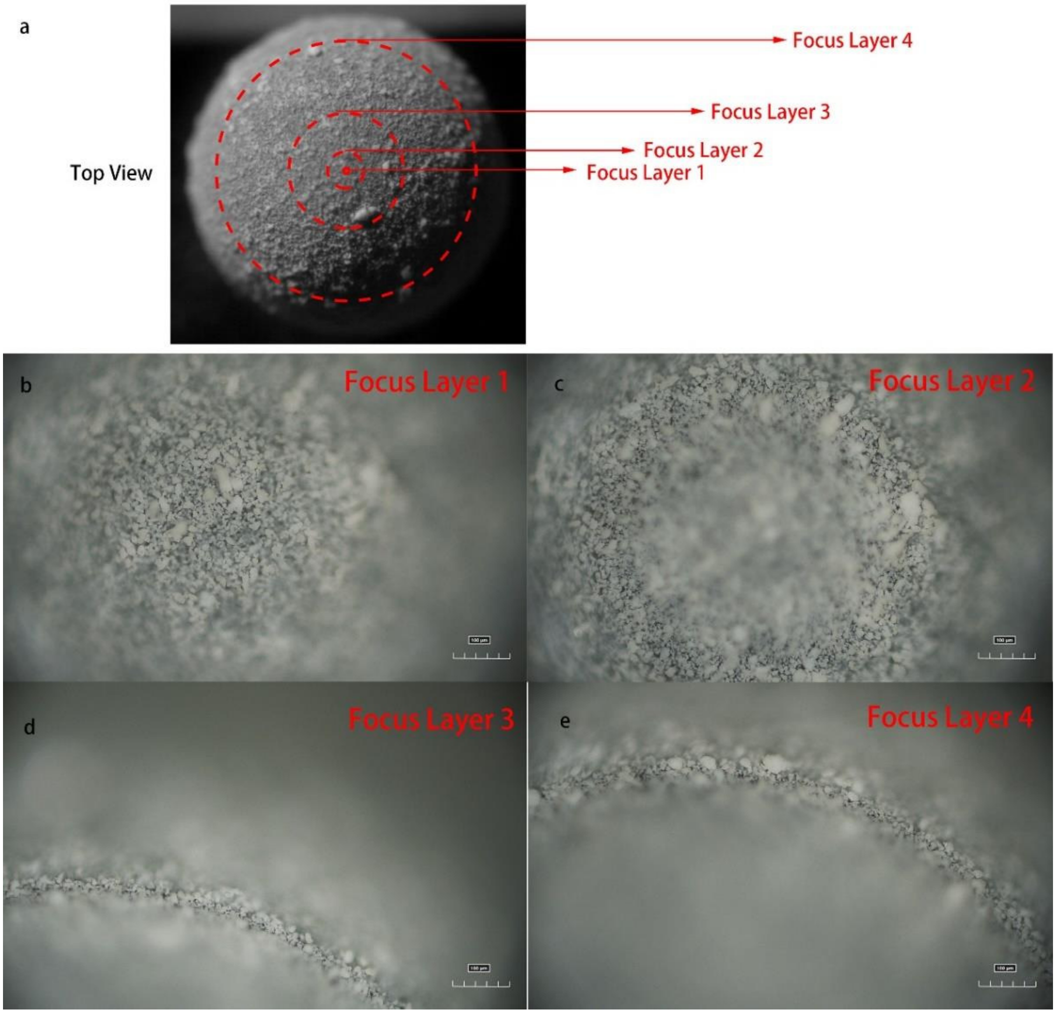

Figure S13 The surface morphology of a LM at different focus layers. The shell of the LM is composed with uniform multilayers of SiO_2 powder, which are arranged compactly on the spherical shell.
